# Effects of the Interaction of Salinity and Rare Earth Elements on the Health of *Mytilus galloprovincialis*: The Case of Praseodymium and Europium

**DOI:** 10.3390/jox14040108

**Published:** 2024-12-18

**Authors:** Carla Leite, Tania Russo, Gianluca Polese, Amadeu M. V. M. Soares, Carlo Pretti, Eduarda Pereira, Rosa Freitas

**Affiliations:** 1 Department of Biology & CESAM, University of Aveiro, 3810-193 Aveiro, Portugalasoares@ua.pt (A.M.V.M.S.); 2 Department of Biology, University of Naples Federico II, 80126 Napoli, Italy; tania.russo@unina.it (T.R.); gianluca.polese@unina.it (G.P.); 3 Department of Veterinary Sciences, University of Pisa, San Piero a Grado, 56122 Pisa, Italy; 4 Interuniversity Consortium of Marine Biology of Leghorn “G. Bacci”, 57128 Livorno, Italy; 5 Department of Chemistry & LAQV-REQUIMTE, University of Aveiro, 3810-193 Aveiro, Portugal

**Keywords:** rare earth elements, salinity, biochemistry, histopathology, adult mussels, sperm

## Abstract

The growing use of products containing rare earth elements (REEs) may lead to higher environmental emissions of these elements, which can potentially enter aquatic systems. Praseodymium (Pr) and europium (Eu) are widely used REEs with various applications. However, their ecotoxicological impacts remain largely unexplored, with poorly understood risks to wildlife. Moreover, organisms also face environmental stressors like salinity fluctuations, and the nature of the interaction between salinity variations and contaminants is not yet clear. Therefore, this study aimed to evaluate the influence of salinity shifts on the impacts of Pr and Eu on adult mussels and the sperm of the species *Mytilus galloprovincialis* after 28 days and 30 min of exposure, respectively. To do so, biochemical and histopathological alterations were evaluated in adults, while biochemical and physiological changes were analysed in sperm. Additionally, the Integrated Biological Index (IBR) was calculated to understand the overall impact of each treatment. The results showed that adult mussels were most affected when exposed to the combination of high salinity and each element, which altered the behaviour of defence mechanisms causing redox imbalance and cellular damage. On the other hand, sperm demonstrated sensitivity to specific REE-salinity combinations, particularly Pr at lower salinity and Eu at higher salinity. These specific treatments elicited changes in sperm motility and velocity: Pr 20 led to a higher production of O_2_^−^ and a decrease in velocity, while Eu 40 resulted in reduced motility and an increase in irregular movement. At both lower and higher salinity levels, exposure to Eu caused similar sensitivities in adults and sperm, reflected by comparable IBR scores. In contrast, Pr exposure induced greater alterations in sperm than in adult mussels at lower salinity, whereas the reverse was observed at higher salinity. These findings suggest that reproductive success and population dynamics could be modulated by interactions between salinity levels and REE pollution, highlighting the need for further investigation into how REEs and environmental factors interact. This study offers valuable insights to inform policymakers about the potential risks of REE contamination, emphasising the importance of implementing environmental regulations and developing strategies to mitigate the impact of these pollutants.

## 1. Introduction

Rare earth elements (REEs) have traditionally been regarded as posing minimal environmental risk due to their low solubility in water and limited (bio)availability. This is because they readily precipitate or form complexes with hydroxides, carbonates, fluorides, phosphates, and humic and fulvic acids [1,2]. For instance, REE-phosphate products have a solubility as low as 10^−25^ mol^2^/L^2^ [2]. However, over the last two decades, due to their unique magnetic, optical, catalytic, and phosphorescent properties, there has been a growing demand for REEs for use in new technologies, such as in equipments in the automotive industry and in renewable energy generation systems [3,4,5]. Among REEs are praseodymium (Pr) and europium (Eu). Praseodymium has been used in permanent magnets to enhance magnetic strength, stability, and energy efficiency [6]; as a contrast agent in medical imaging [7]; and in alloys to improve strength and corrosion resistance [8]. Additionally, this element is used in catalysts to enhance redox reactions and oxygen storage [9] and as a coloring agent in ceramics and glass additives [10]. Despite being one of the rarest REEs, Eu plays an essential role in several industries due to its phosphorescent properties. This element is widely used in phosphors [11], optics, fluorescent lamps, displays, lasers, and light-emitting diodes [11,12]. Additionally, the ability of Eu to absorb neutrons makes it essential in control rods for nuclear reactors [13].

The intensified use of products containing REEs can lead to increasing emissions into the environment. Product manufacturing and technological processes are known to elevate environmental REEs concentrations [14,15]. These elements can reach aquatic systems through various pathways, including atmospheric deposition, surface runoff, and industrial effluents. While atmospheric deposition can occur, it has not been shown to contribute significantly to contamination outside mining regions. In contrast, industrial effluents containing REEs can be directly discharged into surface waters [16]. Praseodymium and Eu have been detected in different aquatic systems. In São Domingos Creek (Portugal), located near to a mining area, measured concentrations range from below the detection limit (<1.0 μg/L) to 51.3 μg/L for Pr and 12.7 μg/L for Eu [15]. In Ría de Huelva (Spain), the maximum concentrations reported were 5.16 μg/L for Pr and 1.48 μg/L for Eu [17]. In the Guadiamar aquifer (Spain), impacted by acid mine drainage, Olías et al. [18]) observed a maximum Pr concentration of 10.21 μg/L in July 2001 and 13.31 μg/L in July 2002. Similarly, Atinkpahoun et al. [19] recorded a maximum Eu concentration of 14.3 µg/L in urban wastewater from the city of Cotonou (Benin, West Africa). A study by Hatje et al. [20] recorded rising REE concentrations in San Francisco Bay (USA) from 1996 to 2013. For instance, Pr concentrations increased from 10.1 to 60.1 pmol/kg, and those for Eu rose from 6.45 to 16.1 pmol/kg [20].

The release of these elements into aquatic systems has led to increased wildlife exposure. However, studies regarding their toxicity in marine and coastal organisms are scarce compared to those for freshwater environments, underscoring a significant knowledge gap. Additionally, there are no specific regulatory restrictions for REEs [16], likely due to the historical perception of minimal risk and a lack of sufficient studies on their environmental impact. This highlights the need for further investigation to better understand the potential risks and ecological implications of REEs in marine ecosystems [16] and provide data for the implementation of regulatory restrictions. A study conducted by Leite et al. [21] showed that the exposure of mussels to Pr (0, 10, 20, 40, and 80 µg/L) led to an increase in metabolic activity and enhanced the activity of antioxidant and biotransformation enzymes, being insufficient to prevent cellular damage. Similarly, the same authors observed that the tested Eu concentrations (0, 10, 20, 40, and 80 µg/L) caused cellular damage in mussels, despite the increase in biotransformation enzyme activity. Histopathological analysis revealed that mussels could not recover from exposure to either element, with lower concentrations causing higher damage to digestive tubules [21]. Markich et al. [22] evaluated the chronic no (significant)-effect concentrations (N(S)ECs) and median-effect concentrations (EC_50_s) induced by Pr and other REEs (yttrium (Y), lanthanum (La), cerium (Ce), neodymium (Nd), gadolinium (Gd), dysprosium (Dy), and lutetium (Lu)) in 30 marine species. The authors observed that for the most sensitive species (a species of sea urchin), the N(S)EC of Pr was 1.9 µg/L and the EC_50_ was 14.4 µg/L, while for the least sensitive (a cyanobacterium), the N(S)EC of Pr was 469 µg/L and the EC_50_ was 3000 µg/L. Overall, Pr, together with La, was the least toxic element among the tested REEs. Similarly, Kurvet et al. [23] reported an EC_50_ of 12.17 mg/L for Pr in the marine bacteria *Vibrio fischeri* in terms of inhibition of luminescence. The authors observed that among the REEs tested (Pr, Ce, Gd, La, and Nd), Pr is ranked second last in terms of toxicity, with La being the least toxic. Tai et al. [24] observed that exposure of the microalgae *Skeletonema costatum* to 29.16 ± 0.61 μmol/L of Eu resulted in a 50% reduction in the growth of algae compared to the controls after 96 h (96 h-EC_50_). Trifuoggi et al. [25] reported that all tested concentrations of Eu (15.2, 152.0, and 15,196.4 μg/L), did not cause significant developmental defects in the early life stages of the sea urchin *Sphaerechinus granularis*. However, while the lower concentrations of Eu (15.2, 152.0 μg/L) similarly did not affect the early development of the sea urchin *Arbacia lixula*, the highest concentration (15,196.4 μg/L) induced significant developmental defects.

Besides pollution, aquatic organisms also face environmental challenges related to climate change. Among the challenges, salinity is one of the key abiotic factors with the power to affect the survival and adaptability of organisms [26]. Salinity variations often result from extreme weather events, with factors like temperature rise and intensification of global rainfall and evaporation cycles contributing to salinity fluctuations in aquatic environments [27]. These changes create differences in osmotic pressure between the surrounding environment and an organism’s tissue cells, leading to stress, including osmoregulation, bioenergetic, and biochemical alterations [28,29,30]. Salinity variations can affect aquatic species, particularly calcifying organisms such as bivalves [31], affecting shell formation, respiration rate, filtration rate, osmoregulation, metabolism, and the oxidative and immune systems [31,32,33,34,35,36,37,38,39]. Moreover, shifts in salinity can not only influence organisms but also alter the behaviour and toxicity of contaminants within aquatic ecosystems. Recent studies showed the influence of salinity shifts on the impacts of REEs. For instance, Andrade et al. [40] showed that lower salinity influenced the effects of La by increasing metabolism, the activity of the defence system, and lipid peroxidation. Another study demonstrated that lower salinity intensified the effects of Y on mussels by increasing their defence mechanisms and neurotoxicity [41]. Nevertheless, the data regarding the organisms’ responses to the combination of REEs and salinity shifts are still scarce.

Considering that organisms in the environment are simultaneously subjected to multiple stressors, this study aimed to investigate the toxicological effects of Pr and Eu (10 μg/L) on *M. galloprovincialis* adults and sperm under varying salinity levels: low (a salinity of 20), control (a salinity of 30), and high (a salinity of 40). While recent research has already highlighted the impacts of Pr and Eu on *M. galloprovincialis*, to our knowledge, there is no available information regarding how salinity influences these effects. *M. galloprovincialis* was chosen as a model species due to its significant economic and ecological value as well as its effectiveness as a bioindicator species. Its widespread distribution, feeding habits, high stress tolerance and the ease with which it can be collected and handled make it an ideal subject for study [42].

## 2. Materials and Methods

### 2.1. Sampling and Experimental Conditions

In the present study, two experiments were performed, one with *Mytilus galloprovincialis* adult mussels that were collected in the Ria de Aveiro coastal lagoon (Portugal) in October 2021 (shell length: 64 ± 3.4 mm; width: 37 ± 2.1 mm) and the other with *M. galloprovincialis* sperm from adults collected in the Gulf of La Spezia (Italy) in May 2022 (shell length: 55 ± 5 mm; width: 28 ± 2.5 mm). While the adult mussels used in both experiments originated from two different locations, previous studies have shown that adults from these sites respond similarly under standard conditions [43,44].

In both experiments, the tested treatments were CTL 20 (non-contaminated seawater at salinity of 20 in order to simulate rainy periods); CTL 30 (non-contaminated seawater at salinity of 30, used as a control salinity); CTL 40 (non-contaminated seawater at salinity of 40 to simulate drought periods); Pr 20 (contaminated seawater with 10 µg/L of Pr at salinity of 20); Pr 30 (contaminated seawater with 10 µg/L of Pr at salinity of 30); Pr 40 (contaminated seawater with 10 µg/L of Pr at salinity of 40), Eu 20 (contaminated seawater with 10 µg/L of Eu at salinity of 20); Eu 30 (contaminated seawater with 10 µg/L of Eu at salinity of 30); and Eu 40 (contaminated seawater with 10 µg/L of Eu at salinity of 40).

The concentration chosen was based on the values found in contaminated environments by Gomes et al. [15] (<1.0–51.3 μg/L for Pr and <1.0–12.7 μg/L for Eu), Olías et al. [18] (maximum Pr concentration of 10.24 μg/L), and Atinkpahoun et al. [19] (maximum Eu concentration of 14.3 µg/L). In addition, the Pr and Eu concentration was also selected since it was the lowest to cause negative effects in mussels within a range of 0–80 μg/L of Pr and Eu in the study by Leite et al. [21]. Given that salinity levels in Ria de Aveiro range from 5 to 15 during winter and from 36 to 37 in summer [45], salinities of 20 ± 1 and 40 ± 1 were chosen to simulate rainy and drought periods, respectively.

### 2.2. Adults’ Exposure

After collection, mussels were transported to the laboratory, where they were held in tanks for a one-week depuration period under control conditions: temperature of 17 ± 1 °C; salinity of 30 ± 1; pH of 8.0 ± 0.1; and natural photoperiod (10 h light/14 h dark). Artificial seawater was prepared by dissolving a commercial salt (RED SEA SALT, Éilat, Israel) in deionized water, with continuous aeration and bi-daily water renewal. Following depuration, mussels were acclimated for an additional week under similar conditions, except with regard to salinity. During this week, mussels were separated into three tanks with adjusted salinity levels: in one tank, the salinity was gradually reduced to 20 at a rate of 2 units per day; in another tank, the salinity was gradually increased to 40 at a rate of 2 units per day; and in the third tank, the salinity was maintained at 30. During the acclimation phase, mussels were fed Algamac protein plus (AquaFauna Bio-Marine, Inc., Hawthorne, CA, USA) at a concentration of 150,000 cells per animal per day.

During the 28-day experiment, a total of 135 mussels were subjected to the previously mentioned treatments. The organisms were placed in different aquaria, each holding 3 L of artificial seawater, with five mussels per aquarium and three aquaria for each treatment. Mussels were fed the same diet used during acclimation three times per week. Temperature, pH, and photoperiod were kept consistent with the depuration/acclimation conditions. Water was renewed weekly, and Pr and Eu concentrations were re-established. Immediately after spiking, water samples were collected from each aquarium to verify the actual concentrations of these elements. In addition, two extra aquaria (blanks) for each contaminated treatment, without organisms, were set up to assess the stability of the elements. Water samples from these aquaria were collected at intervals (0, 72, 120, and 168 h after the spiking) during the first week.

At the end of the exposure period, three mussels were taken from each aquarium (nine per treatment) for biochemical analysis. Each mussel’s tissue was homogenized with a mortar and a pestle under liquid nitrogen and then divided into five 0.5 g fresh-weight (FW) aliquots. The remaining tissue of one of these mussels per aquarium (three per treatment) was used to quantify the concentrations of both elements. In addition, one mussel was collected from each aquarium (three per treatment) for histopathological analysis. For that, each mussel’s tissue was fixed in Davidson solution (a mixture of glycerol, formalin, 95% ethanol, and seawater) for a period of 24 h.

#### 2.2.1. Praseodymium and Europium Quantification

The concentrations of Pr and Eu in water and mussels’ tissues were measured using inductively coupled plasma mass spectrometry (ICP-MS). Both elements had a quantification limit (LOQ) of 0.01 µg/L. Upon collection, water samples were acidified with nitric acid (HNO_3_) (65%), and before the analysis, the samples were diluted by a factor of 20 to reach salinity < 2.

Mussels’ tissue samples were freeze-dried, and 0.2 g of each sample was digested with an acid mixture containing 1 mL of HNO_3_ 65% (*v*/*v*), 2 mL of hydrogen peroxide (30%), and 1 mL of ultrapure water (H_2_O). Digestion was carried out in a CEM MARS 5 microwave, where the temperature was ramped up to 180 °C over 15 min and maintained at this level for an additional 5 min. Following digestion, ultrapure H_2_O was added to each sample to reach a final volume of 25 mL. To ensure quality control, an analysis of blank samples (with no tissue) was conducted, showing concentrations below the LOQ. The certified reference material (CRM) BCR-668 (Mussel Tissue: 12.3 ± 1.1 µg/kg Pr and 2.79 ± 0.16 µg/kg Eu) was analysed, and the percentage of recovery for Pr was 87%. The results were validated with this acceptable recovery percentage for Pr since the concentration of Eu in the CRM was below the LOQ. Differences between duplicates were <1%.

#### 2.2.2. Biochemical Analysis

To assess the performance of the adult mussels, the biochemical parameters analysed were associated with energy balance (electron transport system (ETS) activity, total protein (PROT) and glycogen (GLY) contents); antioxidant and biotransformation defences (such as superoxide dismutase (SOD), catalase (CAT), glutathione peroxidase (GPx), carboxylesterases with *p*-nitrophenyl acetate (CbEs-*p*NPA), carboxylesterases with *p*-nitrophenyl butyrate (CbEs-*p*NPB), glutathione S-transferases (GSTs) activities); redox status and indicators of cellular damage (reduced and oxidized glutathione ratio (GSH/GSSG), lipid peroxidation (LPO), and protein carbonylation (PC) levels). Detailed procedures for the extractions and methods can be found in studies published by De Marchi et al. [43], Andrade et al. [44], and Leite et al. [46].

#### 2.2.3. Histopathological Analysis

The histopathological alterations were evaluated in the gills and digestive tubules. After the fixation in Davidson’s solution for 24 h, the mussels’ tissues were transferred to 70% ethanol, and the gills and digestive gland were dissected. Dehydration and embedding procedures were then conducted according to the methods described by Leite et al. [47]. For staining, a haematoxylin solution was applied following the method described by Leite et al. [46]. Tissue alterations were observed under an optical microscope at 40× magnification, and histopathological indices (*I_h_*) were calculated as outlined by Leite et al. [46].

### 2.3. Sperm Exposure

Mussels underwent a two-week depuration and acclimation process using filtered natural seawater (0.45 µm). The organisms were maintained at a temperature of 17 ± 1 °C, with a natural photoperiod. Initially, the salinity matched that at the collection site (38), and it was then gradually reduced over the first week to reach a salinity of 30. Throughout the second week, a salinity of 30 was consistently maintained as the control salinity. The seawater was aerated continuously and replaced every two days. During the second week, mussels were provided with the same diet used during the experience with the adult mussels.

The collection of the sperm was carried out by slightly cutting the mantle following the procedure outlined by Mikhailov et al. [48]. For each parameter, a sperm suspension pool from three mussels was used, with both morphology and motility checked beforehand. The concentration in each pool (cells/mL) was measured using an Olympus CH-2 optical microscope at 20× magnification and a Bürker counting chamber. After determining the initial concentration, these pools were diluted to reach the targeted concentration for each parameter. The sperm suspensions were then incubated for 30 min and subjected to the treatments mentioned before (contaminant 1:99 sperm suspension). After incubation, the suspensions were centrifuged at 3000× *g* at 4 °C for 5 min, and the resulting pellets were resuspended in filtered natural seawater at salinities of 20, 30, or 40, depending on the treatment.

#### Biochemical and Physiological Analysis

The quality of *M. galloprovincialis* sperm cells was assessed by analysing biochemical parameters related to the production of reactive oxygen species (ROS), specifically superoxide anion (O_2_^−^) and hydrogen peroxide (H_2_O_2_), as well as an indicator of cellular damage, such as LPO levels. These parameters are described in Leite et al.’s work [47].

Additionally, sperm quality was also evaluated through physiological parameters, such as the percentage of motility (% MOT), curvilinear velocity (VCL), linearity (LIN), and wobble (WOB). The methodologies for assessing these parameters are detailed in Leite et al.’s work [47].

### 2.4. Data Analyses

#### 2.4.1. Bioconcentration Factor

The bioconcentration factor (BCF) was calculated following the approach by Arnot and Gobas [49]. This method can be used to calculate the ratio of the concentrations in the tissue to the concentration in the exposure medium, measured immediately after spiking.

#### 2.4.2. Statistical Analysis

A non-parametric permutational analysis of variance (PERMANOVA + add-on in PRIMER v6) [50] was applied to examine the concentrations of Pr and Eu in the mussel tissue and several biological responses. These responses included biochemical changes in adult mussels, such as in ETS, PROT, GLY, SOD, CAT, GPx, CbEs-*p*NPA, CbEs-*p*NPB, GSTs, GSH/GSSG, LPO, and PC, as well as histopathological indices (*I_h_*_Digestive tubules_ and *I_h Gills_*). Additionally, biochemical and physiological changes in sperm were also included (ROS (O_2_^−^), ROS (H_2_O_2_), LPO, % MOT, VCL, LIN, and WOB). Pairwise comparisons were conducted when the main test showed significant differences (*p* < 0.05). The null hypotheses tested were as follows: (i) in terms of concentrations in mussel tissue, no significant differences will be observed among salinities (20, 30, 40) (in the table, significant differences are denoted by different bold and italic lowercase letters for Pr and Eu concentrations in CTL treatments, different uppercase letters for Pr concentrations in Pr treatments, and different lowercase letters for Eu concentrations in Eu treatments); (ii) in terms of concentrations in mussel tissue, no significant differences will be observed between CTL and Pr as well as CTL and Eu at each salinity (20, 30, 40) (significant differences are labelled in the table, with the *p*-value in bold); (iii) in terms of biological responses, no significant differences will be observed among salinities (20, 30, 40) in mussels and sperm (in the figures, significant differences are denoted by different bold and italic lowercase letters for CTL, different uppercase letters for Pr, and different lowercase letters for Eu); (iv) in terms of biological responses, no significant differences will be observed between CTL, Pr, and Eu at each salinity (20, 30, 40) in mussels and sperm (significant differences are labelled in the table, with the *p*-value in bold).

#### 2.4.3. Principal Coordinates Analysis

The biological responses (biochemical and histopathological changes in adults as well as biochemical and physiological changes in sperm) were subjected to ordination analysis by Principal Coordinates (PCO) analysis to determine the relationship between treatments. The Euclidean distance similarity matrix was used to calculate the distance between centroids among the different treatments (CTL 20, 30, and 40; Pr 20, 30, and 40; and Eu 20, 30, and 40). Additionally, vectors relating to the biological descriptors were overlaid on the PCO graph using Spearman’s correlation, with a correlation threshold higher than 70%.

#### 2.4.4. Independent Action Model

The type of interaction in each biological response between the combined stressors (Pr 20, Eu 20, Pr 40, and Eu 40) was classified using the Independent Action (IA) model, as detailed by Song et al. [51]. Predicted effects for these combinations were calculated for each biological response according to the method reported by Leite et al. [52]. To confirm the additivity assumptions, observed combination responses (Pr 20, Eu 20, Pr 40, Eu 40) were compared with the IA model predictions. If the predicted values fell outside the 95% confidence intervals (based on the t-distribution) of the observed responses, the effects were assumed to be non-additive [53]. In such cases, the type, strength, and direction of these interactions were evaluated using the methodology proposed by Piggott et al. [53] and the terminology described by Delnat et al. [54].

#### 2.4.5. Integrated Biological Response Index

The overall response of adults and sperm to the treatments at salinities of 20 and 40 was evaluated by calculating the Integrated Biological Response Index version 2 (IBRv2), following the methods reported by Beliaeff and Burgeot [55] with adaptations by Sanchez et al. [56] and described in detail by Leite et al. [52]. Toward this end, the deviation of the stress treatments (CTL 20, Pr 20, Eu 20, CTL 40, Pr 40, and Eu 40) from the CTL treatment (CTL 30) was calculated. The IBRv2 index was determined and the final score was divided by the number of parameters for adults and sperm. The overall response provided by the IBRv2 index was discussed in terms of the generated scores, with higher IBRv2 values showing a higher degree of responsiveness by mussels/sperm.

## 3. Results and Discussion

This study aimed to investigate the interaction between different salinity levels and praseodymium (Pr) and europium (Eu), assessing their effects on *Mytilus galloprovincialis*. To achieve this, biochemical, histopathological, and physiological responses were evaluated in adults and sperm. Additionally, the Independent Action (IA) model was used to characterize the type of interaction for each observed effect between the elements and the different stress salinities, since it is commonly used for combinations of contaminants and natural stressors [54]. The IA model was calculated for four combinations: Pr 20, Eu 20, Pr 40, and Eu 40. The results indicated that for each treatment except for Pr 40, half of the responses were additive (Table 1, Table 2, Table 3 and Table 4). This suggests that, for a significant portion of the responses, the combined effects of the elements and salinity did not significantly alter the mussels’ response beyond what would be expected by summing the individual effects of each factor. Furthermore, at the lowest salinity level, Pr showed a higher synergetic potential than Eu (Table 1 and Table 2), while at the highest salinity level, Pr induced the highest number of antagonistic interactions (Table 3 and Table 4), suggesting that this salinity level contributed more to mitigate the effects of Pr compared to Eu. Excluding the additive responses, most of the mussels’ responses to Eu were antagonistic (Table 2 and Table 4), suggesting that, in general, the effects of Eu were often neutralized by salinity conditions.

**Figure 1:**
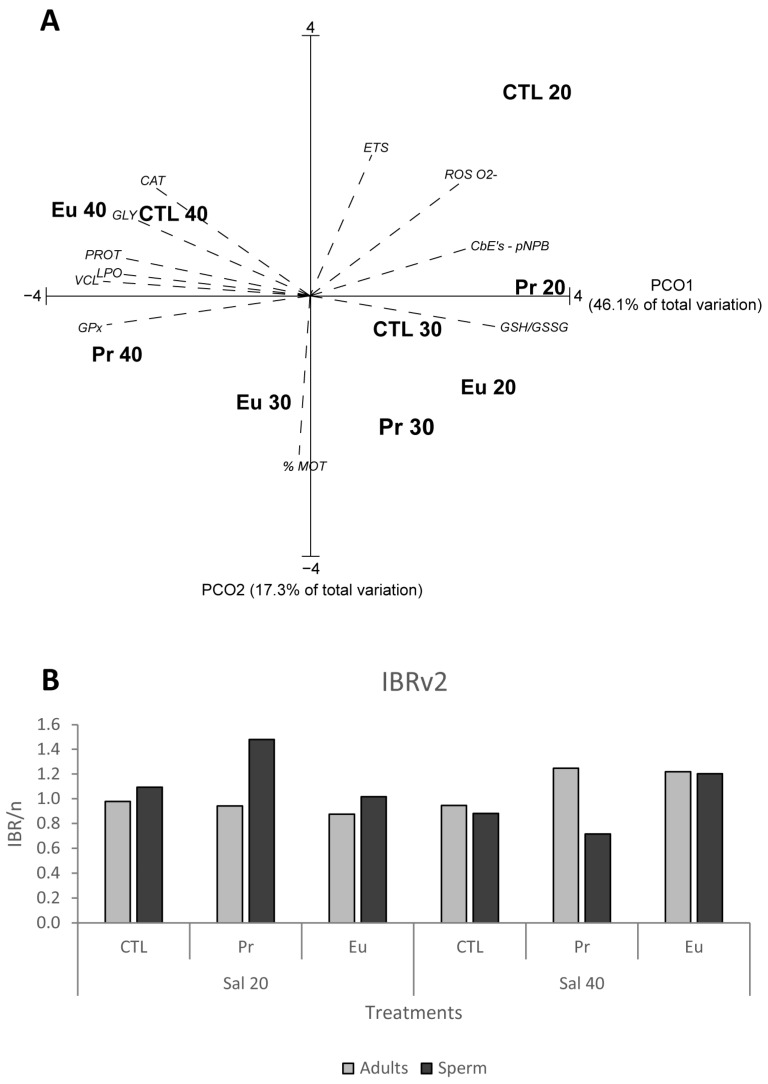
(**A**) Centroid ordination diagram (PCO) based on all biological responses measured in adults and sperm of *Mytilus galloprovincialis* exposed to control (CTL), praseodymium (Pr), and europium (Eu) at salinities of 20, 30, and 40. Spearman correlation vectors were superimposed as supplementary variables (r > 0.7): ETS, PROT, GLY, CAT, GPx, CbEs-pNPB, GSH/GSSG, LPO (adults), O_2_^−^, % MOT, and VCL; (**B**) Integrated Biological Response (IBRv2) score divided by the number of parameters measured in adults and sperm of *Mytilus galloprovincialis* exposed to control (CTL), praseodymium (Pr), and europium (Eu) at salinities of 20 and 40.

To determine the similarities and distances between the treatments, Principal Coordinates (PCO) analysis was employed. This method also enables the visualisation of the relationships and patterns between treatments, and, in the case of this study, PCO separated the salinity of 40 from the salinity of 20 (Figure 1A). This division shows the influence of salinity on the observed responses. Based on this, the following sections will focus on evaluating the influence of both salinities in the effects of Pr and Eu.

### 3.1. The Influence of Lower Salinity on the Elements’ Effects

#### 3.1.1. Adults’ Exposure

##### Praseodymium and Europium Concentration in Water and Mussel Tissue

The data regarding the concentrations of both elements in water after spiking can be found in Table 5, and the concentrations over one week, regarding the stability of the elements, are presented in Table 6. The data indicate that lower salinity may have distinct influences on reductions in Pr and Eu levels. At lower salinity, the Pr levels in water decreased by 25% after one week, which is lower than 31% at the control salinity. For Eu, at lower salinity, the loss was 8%, which is higher than the loss at the control salinity (3%). These results show the differential sensitivity of these elements to changes in salinity, likely driven by their unique chemical properties.

The bioconcentration factor (BCF) showed that the accumulation of both elements by mussels was higher at a salinity of 20, despite the fact that the concentration of Eu in mussel tissue did not significantly differ from that at the other salinities (Table 5). This could be due to a reduced concentration of common ions and anions, which decreases competition for absorption and limits the formation of complexes, while increasing the bioavailability of the elements. In addition, the accumulation of Pr at a salinity of 20 was higher than that of Eu at the same salinity (Table 5). This could have occurred because, overall, Eu has a smaller ionic radius and a higher charge density than Pr due to the lanthanide contraction. Because of this, Eu can form stronger bonds, which may reduce its solubility and lead to precipitation or the formation of less soluble complexes [16]. Other rare earth elements (REEs), namely, lanthanum (La) and gadolinium (Gd), also exhibited an increased accumulation in mussels with a decrease in salinity [40,44]. In line with our findings, these studies reported that Gd, which has a smaller ionic radius and a higher charge density than La, also showed lower accumulation levels than La, highlighting the influence of ionic size and charge on metal bioavailability and uptake.

##### Biochemical Analyses

**Figure 2:**
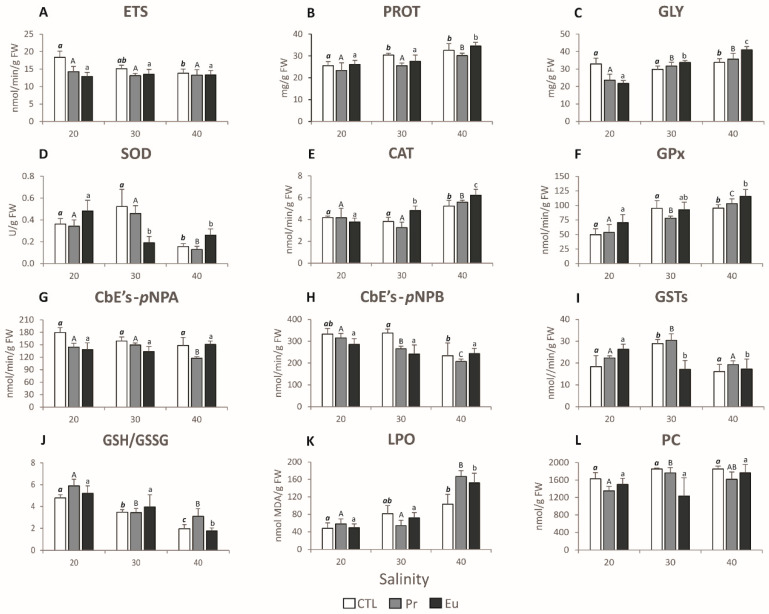
(**A**) Electron transport system (ETS) activity, expressed in nmol per min per g of fresh weight (FW); (**B**) protein (PROT) content, expressed in mg per g of FW; (**C**) glycogen (GLY) content, expressed in mg per g of FW; (**D**) superoxide dismutase (SOD) activity, expressed in U per g of FW, where U is a reduction of 50% in nitroblue tetrazolium (NBT) levels; (**E**) catalase (CAT) activity, expressed in nmol of formaldehyde per min per g of FW; (**F**) glutathione peroxidase (GPx) activity, expressed in nmol per min per g of FW; (**G**) carboxylesterase with *p*-nitrophenyl acetate (CbE-*p*NPA) activity, expressed in nmol per min per g of FW; (**H**) carboxylesterases with *p*-nitrophenyl butyrate (CbEs-*p*NPB), expressed in nmol per min per g of FW; (**I**) glutathione S-transferases (GSTs) activity, expressed in nmol per min per g of FW; (**J**) reduced-to-oxidized glutathione (GSH/GSSG) ratio; (**K**) lipid peroxidation (LPO) levels, expressed in nmol of malondialdehyde (MDA) per g of FW; (**L**) protein carbonylation (PC) levels, expressed in nmol per g of FW, in *Mytilus galloprovincialis* exposed to control (CTL), praseodymium (Pr), and europium (Eu) at salinities of 20, 30, and 40 for 28 days. Results are means with standard deviations. Significant differences (*p* < 0.05) among salinities are denoted by different letters (bold italic lowercase letters stand for CTL treatments, uppercase letters stand for Pr treatments, and lowercase letters stand for Eu treatments).

To assess the energy balance of the mussels, their metabolic capacity was measured through electron transport system (ETS) activity, while energy reserves were determined by analysing glycogen (GLY) and protein (PROT) contents [57,58,59]. The ETS activity displayed a positive correlation with CTL 20 in the PCO graph (Figure 1A), and indeed the results indicated that low salinity alone induced a slight metabolic increase compared to the CTL 30 (Figure 2A). This increase in metabolism may be linked to an enhanced filtration rate, as salinity is an important abiotic factor influencing the filtration rate in bivalves [35]. For instance, Guzmán-Agüero et al. [33] observed that reduced salinity led to an increase in the filtration rate in the bivalve species *Crassostrea corteziensis.* The rise in metabolism could also suggest that mussels might be using the additional energy to boost their defence mechanisms. In addition, Pr seemed to consistently suppress metabolism, which was observed at the control and lower salinity (Table 7). Such a response can indicate that Pr plays a dominant role in terms of metabolic capacity, as predicted by the IA model. In the case of Eu, although no significant changes were observed in the mussels’ metabolism at the control salinity, exposure to lower salinity resulted in metabolic depression (Table 7). This suppression of metabolic activity in the mussels exposed to Pr and Eu at lower salinity may suggest a potential behavioural response, such as the reduction in filtration rate caused by prolonged valve closure. This behaviour was already documented by Gosling [60] and Ortmann and Grieshaber [61] when bivalves were placed under stressful conditions. It is important to note that the slight metabolic increase observed in the mussels exposed to low salinity alone was not observed in those exposed to the elements at low salinity. This might suggest that the presence of these elements interfered with the mussels’ ability to adapt to low-salinity stress. Accompanying the higher metabolic capacity of the mussels exposed to low salinity alone, lower PROT content was observed compared to CTL 30 (Figure 2B), indicating that the organisms were probably using their protein reserves to enhance their defence mechanisms under osmotic stress. In contrast, despite the metabolic suppression observed in the mussels exposed to Pr 20 and Eu 20, both groups showed a significant use of GLY content (Figure 2C and Table 7). This explains the negative correlation between these treatments and GLY in the PCO graph (Figure 1A). The organisms were likely using GLY to fuel up their defence mechanisms. The utilisation of GLY in these treatments points to a synergetic response (Table 1 and Table 2), suggesting that the mussels were under significant stress and that the closure of the valves was not enough. As a result, the mussels needed additional energy to enhance the activity of their defence mechanisms. Similarly, Andrade et al. [40] showed that non-contaminated mussels at a salinity of 20 had increased metabolism and decreased PROT content compared to non-contaminated mussels at a salinity of 30. Andrade et al. [41] further demonstrated that mussels exposed to yttrium (Y) at a salinity of 20 maintained their metabolic capacity similar to those Y-exposed at a salinity of 30.

To prevent cellular damage, organisms possess defence mechanisms, which include antioxidant and biotransformation enzymes. These two types of enzymes serve distinct functions; while antioxidant enzymes help neutralize the excess of reactive oxygen species (ROS) to prevent oxidative stress, biotransformation enzymes facilitate the detoxification and excretion of pollutants from cells [62]. The data obtained demonstrated that at lower salinity, only the Eu-exposed mussels activated their antioxidant enzymes, specifically superoxide dismutase (SOD), when compared to the Eu-exposed mussels at control salinity (Figure 2D). Regarding biotransformation enzymes, carboxylesterases with *p*-nitrophenyl butyrate (CbEs-*p*NPB) exhibited a positive correlation with Pr 20 (Figure 1A) since this treatment induced a significant increase in that enzyme compared to the Pr-exposed mussels at control salinity (Figure 2H). In addition, Eu 20 induced greater glutathione S-transferases (GSTs) activity compared to that for the Eu-exposed mussels at a salinity of 30 (Figure 2I). These enzymatic activations could explain the increase in GLY consumption observed in these treatments. The activation of the mentioned antioxidant and biotransformation defences at lower salinity was enough to prevent cellular damage, as demonstrated by the lack of lipid peroxidation (LPO) and protein carbonylation (PC) (Figure 2K,L and Table 7). This explains why, in the PCO graph, LPO is positioned opposite to Pr 20 and Eu 20 (Figure 1A). In terms of redox balance assessed using the ratio of reduced to oxidized glutathione (GSH/GSSG) [62], the PCO analysis showed that this parameter has a positive correlation with Pr 20 and Eu 20 (Figure 1A), which aligns with the higher GSH/GSSG values observed in mussels exposed to both treatments (Figure 2J). This suggests that these mussels were able to maintain redox homeostasis. The increase in GSH/GSSG showed that GSH content was being accumulated and not converted in GSSG, which can be related to the decrease in GPx and GSTs activities (Figure 2F,I) since these two enzymes are used for the conversion of GSH into GSSG.

##### Histopathological Analyses

**Figure 3:**
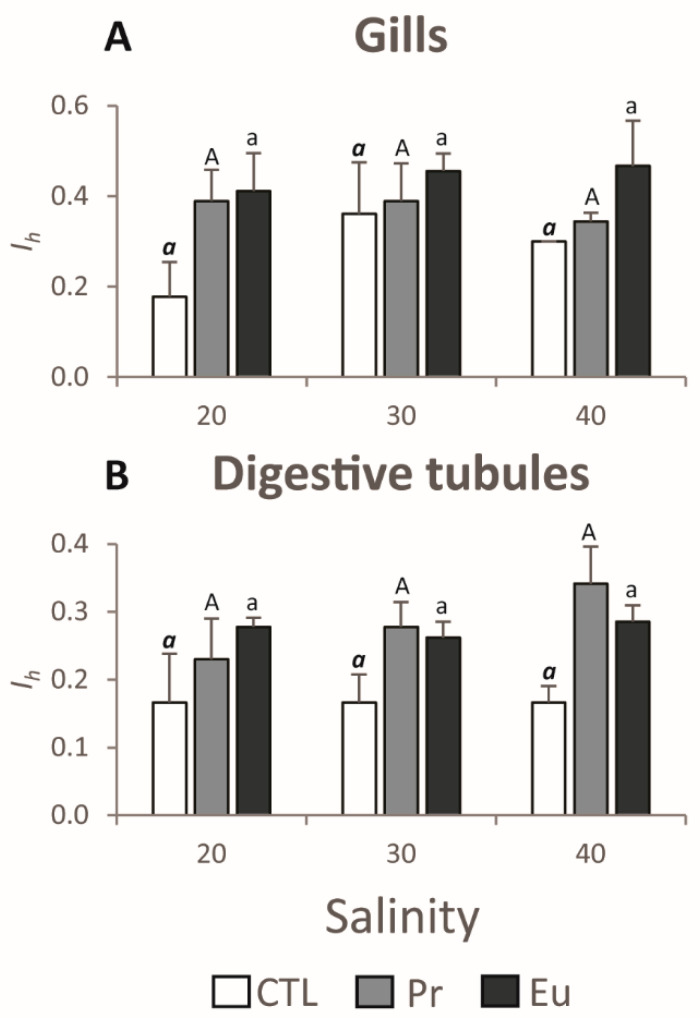
Histopathological index (Ih) in (**A**) gills; (**B**) digestive tubules of *Mytilus galloprovincialis* exposed to control (CTL), praseodymium (Pr), and europium (Eu) at salinities of 20, 30, and 40 for 28 days. Results are means with standard deviations. Significant differences (*p* < 0.05) among salinities are denoted by different letters (bold italic lowercase letters stand for CTL treatments, uppercase letters stand for Pr treatments, and lowercase letters stand for Eu treatments).

**Figure 4:**
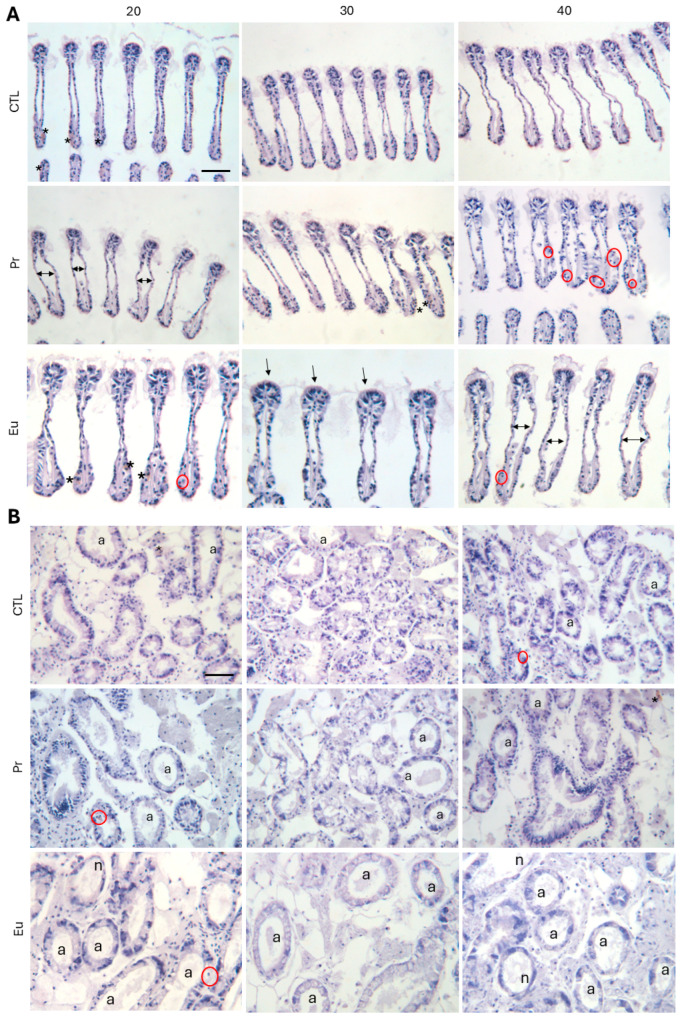
Representative micrographs of the histopathological alterations observed in (**A**) gills and (**B**) digestive tubules of *Mytilus galloprovincialis* exposed to control (CTL), praseodymium (Pr), and europium (Eu) at salinities of 20, 30, and 40 for 28 days and stained with haematoxylin. Alterations: lipofuscin aggregates (*); enlargement of the central vessel (double-headed arrow); haemocyte infiltration (red circle); loss of cilia (single arrow) atrophy (a); necrosis (n). Scale bar: 50 µm.

The gills and digestive gland are critical organs for assessing the health statuses of mussels when exposed to stressors. The gills serve as the initial interface with the surrounding water, filtrating the water before it reaches other internal organs [63,64]. On the other hand, the digestive gland plays a central role in the biotransformation of xenobiotics, functioning as the primary site for detoxification and metabolic processing [65]. This makes both organs highly sensitive indicators of environmental changes and contaminant exposure. The data regarding the histopathological alterations showed that the index (*I_h_*) in the gills of mussels exposed to Pr and Eu at lower salinity was similar to that for those exposed to Pr 30 and Eu 30 (Figure 3A). However, in the presence of these contaminants at a salinity of 20, the *I_h_* was significantly higher compared to that for CTL 20 (Table 7), with higher haemocyte infiltration, as well as an increased number of enlarged vessels (Figure 4A). This shows that the contaminants impacted gills, and it can explain why mussels likely close their valves. The haemocyte infiltration and enlarged vessels can be linked with inflammatory processes [66,67,68]. Concerning the digestive tubules, Pr 20 and Eu 20 showed similar *I_h_* to Pr 30 and Eu 30 (Figure 3B), with the latter displaying significantly higher *I_h_* than CTL 30 (Table 7), characterized by an increase in the number of atrophied tubules and the appearance of necrosis (Figure 4B). This type of injury is associated with inflammatory processes [69] and may contribute to physiological impairments, such as inefficient nutrient absorption [70]. This suggests that these elements induced consistent histopathological injuries, regardless of the salinity level. Furthermore, the absence of significant differences between CTL 20 and CTL 30, combined with the similar *I_h_* values between Pr 20 and Eu 20 to their respective salinity 30 counterparts (Figure 3A,B), indicates that the observed effects are primarily due to the elements rather than salinity stress. Also, Coppola et al. [71] showed that mussels exposed for 28 days to 50 µg/L of mercury (Hg) at a salinity of 20 presented significantly higher *I_h_* in their gills compared to those exposed to CTL at a salinity of 20.

#### 3.1.2. Sperm Exposure

**Figure 5:**
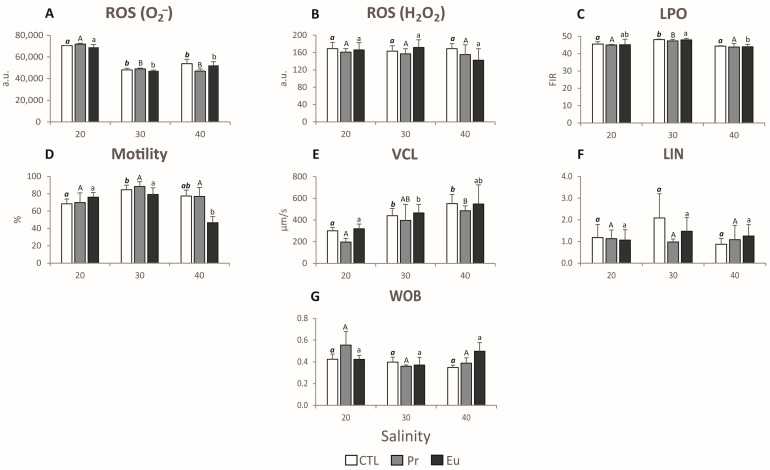
(**A**) Superoxide-anion-derived reactive oxygen species (ROS (O_2_^−^)) production, expressed in arbitrary units of fluorescence intensity (a.u.); (**B**) hydrogen-peroxide-derived reactive oxygen species (ROS (H_2_O_2_)) production, expressed in a.u.; (**C**) lipid peroxidation (LPO) levels, expressed as the fluorescence intensity ratio (FIR); (**D**) motility, expressed as percentage (%) of motility; (**E**) curvilinear velocity (VLC), expressed in µm per s; (**F**) linearity (LIN); and (**G**) wobble (WOB) in sperm of the species *Mytilus galloprovincialis* exposed to control (CTL), praseodymium (Pr), and europium (Eu) at salinities of 20, 30, and 40 for 30 min. Results are means with standard deviations. Significant differences (*p* < 0.05) among salinities are denoted by different letters (bold italic lowercase letters stand for CTL treatments, uppercase letters stand for Pr treatments, and lowercase letters stand for Eu treatments).

The sperm under low-salinity conditions showed a significant increase in the production of superoxide anion (O_2_^−^) compared to the sperm at the control salinity (Figure 5A). This can indicate that salinity played a more significant role than the elements in inducing ROS production. In addition, Pr 20 induced the highest amount of O_2_^−^ production among the treatments at the same salinity (Figure 5A and Table 7). This elevated O_2_^−^ production at lower salinity can indicate that the sperm were under osmotic stress. However, despite this, no increase in H2O2 production was observed, nor was there any LPO (Figure 5B,C and Table 7), which can suggest that the defence mechanisms were activated and sufficient to prevent cell damage. Regarding the motility of the sperm, lower salinity by itself seemed to be prejudicial to the motility of the sperm, as there was a decrease in the percentage of motility at CTL 20 compared to the sperm at CTL 30 (Figure 5D). However, the combination of lower salinity and Pr or Eu did not induce significant differences compared to their counterparts at control salinity (Figure 5D). This may suggest that these elements have a neutralizing effect on the stress response, preventing further motility decline compared to CTL 20. On the other hand, the velocity of the sperm exposed to the treatments at lower salinity was significantly lower than that for the sperm at control salinity (Figure 5E), which explains the opposite position of the velocity from Pr 20 and Eu 20 in the PCO (Figure 1A). Furthermore, the lowest velocity was observed for the Pr-exposed sperm (Figure 5E and Table 7). This behaviour is likely due to the increase in O_2_^−^ production observed in that treatment, since it may affect mitochondrial function and, subsequently, sperm function [72]. Furthermore, despite there being no significant differences (Figure 5F), it seemed that linearity (LIN) decreased in sperm exposed to the treatments at lower salinity compared to that for the sperm at CTL30 (Figure 5F). In addition, a more pronounced irregular movement (WOB) was observed in sperm exposed to Pr 20 (with no significant difference) than in the sperm exposed to CTL and Pr 30 (Figure 5G). Trifuoggi et al. [25] demonstrated that when sperm of two sea urchin species (*Paracentrotus lividus* and *Sphaerechinus granularis*) were exposed to 10^−4^ M and 10^−5^ M of Eu, the offspring of the sperm exposed to 10^−5^ M did not present developmental defects. In contrast, the offspring from the sperm exposed to 10^−4^ M were severely affected, with significant developmental defects. Furthermore, Cuccaro et al. [73] observed that the sperm of *Ficopomatus enigmaticus* exposed to cadmium (Cd), arsenic (As), and zinc (Zn) at a salinity of 20 did not exhibit higher LPO levels than the sperm exposed at a salinity of 30.

### 3.2. The Influence of Higher Salinity on the Elements’ Effects

#### 3.2.1. Adult Exposure

##### Praseodymium and Europium Concentrations in Mussel Tissue

Higher salinity had contrasting effects on Pr and Eu when compared to the control salinity. While Pr loss decreased slightly at a salinity of 40 (amounting to 25% at a salinity of 40 and 31% at a salinity of 30), Eu loss increased dramatically (28% at a salinity of 40 and 3% at a salinity of 30). These findings emphasise the differential behaviour of these elements in saline environments, driven by their distinct chemical properties, such as solubility and interactions with other ions in the system. 

The accumulation of Pr at higher salinity was lower than the accumulation of Pr in mussels exposed to control salinity, while the accumulation of Eu remained similar (Table 5). This is likely because Pr has a higher ionic radius and may experience more ion competition under high-salinity conditions. On the other hand, Eu may form stronger complexes and have more stable bioavailability, explaining the more consistent accumulation across salinities. Despite this, the concentrations of the elements in the mussels’ tissues were more similar at a salinity of 40 compared to a salinity of 30 (Table 5). Similarly, Freitas et al. [74] observed that the concentrations of lead (Pb) in mussels’ tissues were similar at the higher salinity (35) and control salinity (30).

##### Biochemical Analyses

Unlike the mussels at CTL 20, those at CTL 40 slightly decreased their metabolic capacity (Figure 2A), suggesting that they were attempting to reduce filtration by closing their valves to minimize stress. On the other hand, the mussels exposed to Pr and Eu at higher salinity did not significantly change their metabolic capacity compared to those exposed to Pr and Eu at control salinity (Figure 2A). This suggests that these elements could have neutralized the need to close the valves imposed by the higher salinity. Regarding the energy reserves, along with the limited changes in their metabolic capacity, the mussels kept at higher salinity increased their PROT and GLY content compared to those at control salinity (Figure 2B,C), revealing that the mussels were able to avoid energy reserve expenditure under this condition. This explains the association in the PCO graph between the energy reserves and the treatments at a salinity of 40 (Figure 1A). Similarly, Freitas et al. [31] observed that when exposed to a higher salinity (35), this species reduced its metabolism and increased GLY and PROT content compared to those under the control salinity (28). De Marchi et al. [43] also noted a trend of reduced metabolism in mussels at a salinity of 37 compared to those at 28. However, the author also found no changes in the metabolism between mussels exposed to carbon nanotubes at a higher salinity than those also exposed but at the control salinity.

Concerning antioxidant enzymes, the mussels exposed to the highest salinity had higher CAT activity than their counterparts at a salinity of 30 (Figure 2E), which justifies the strong correlation observed in the PCO analysis between this parameter and CTL 40 and Eu 40 (Figure 1A). Mussels exposed to Pr 40 also activated GPx (Figure 2F) to eliminate the excess of ROS, explaining the association observed in the PCO graph between the treatment and this parameter (Figure 1A). Regarding the biotransformation enzymes, the mussels did not activate these defences when they were kept under high salinity (Figure 2G–I and Table 7). It seems that Pr 40 induced the inhibition of these enzymes since the activity decreased compared to that at a salinity of 30 (Figure 2G–I). Nevertheless, the activation of antioxidant enzymes was enough to avoid PC (Figure 2L) but not sufficient to prevent LPO (Figure 2K), justifying the positive association between the treatments at a salinity of 40 and LPO (Figure 1A). Similarly, a study conducted by Andrade et al. [44] noted that mussels exposed to Gd at a salinity of 40 presented significantly more LPO than those exposed to Gd at a salinity of 30. The present study, further demonstrates that the mussels subjected to high salinity were under redox imbalance (Figure 2J). The results revealed that in the PCO graph, GSH/GSSG was in the opposite position from CTL 40 and Eu 40 (Figure 1A), and this is because mussels under these treatments presented the lowest values of GSH/GSSG (Figure 2J). This indicates that these mussels were using more GSH to neutralize ROS and convert GSH to GSSG, likely using GPx activity since the activity of this enzyme increased at this salinity (Figure 2F).

##### Histopathological Analyses

The histopathological data indicated that salinity did not have much influence on the alterations observed in gills and digestive tubules. The *I_h_* in gills and digestive tubules showed no significant differences between the mussels at control salinity and those kept under higher salinity (Figure 3A,B). On the other hand, at a salinity of 40, the mussels exposed to Pr and Eu presented a significantly higher *I_h_* compared to those exposed to CTL (Table 7). In the case of the gills, the mussels exposed to Pr 40 showed higher haemocyte infiltration, while those exposed to Eu presented an increase in lipofuscin aggregates and enlarged central vessels (Figure 4A). In terms of digestive tubules, the Pr-exposed mussels presented more lipofuscin aggregates and an increased number of atrophied tubules, while the Eu-exposed mussels only presented more necrotic tubules (Figure 4B). Like the mussels kept under low salinity, those under high salinity were also under stress since the inflammatory system was likely activated due to haemocyte infiltration in the gills and atrophied digestive tubules. Additionally, lipofuscin aggregates showed that under this salinity, cellular damage was present both in the gills and digestive tubules [75]. Similarly, Coppola et al. [71] observed that after 28 days of exposure to Hg (50 µg/L), mussels of *M. galloprovincialis* kept at a salinity of 40 presented significantly higher *I_h_* in their gills and digestive tubules compared to those at CTL 40. Also, Pagano et al. (2016b) observed histological alterations in the gills and digestive system of *M. galloprovincialis* when exposed to quaternium-15 under high salinity (37) in comparison to non-contaminated mussels at the same salinity.

#### 3.2.2. Sperm Exposure

In the case of the sperm, it seemed that they were not under oxidative stress when exposed to higher salinity, as there was no increase in ROS production compared to the sperm kept under the control salinity, nor was any LPO observed (Figure 5A–C and Table 7). This could have happened because the ROS were being neutralized by the defence mechanisms or because these treatments did not induce sufficient stress to induce oxidative stress. In terms of motility, compared to the sperm at control salinity, the percentage of motile sperm was slightly lower in the non-contaminated sperm at higher salinity and significantly lower in the Eu-exposed sperm (Figure 5D). In addition, at higher salinitya salinity of 40, the Eu-exposed sperm presented a significantly lower percentage than the CTL and Pr-exposed ones at the same salinity (Table 7). This might suggest that Eu may exacerbate the effects of high salinity, as this combination is detrimental to the motility of sperm. On the other hand, the velocity of the sperm at a salinity of 40 was not reduced when compared to that for the sperm kept at a salinity of 30 (Figure 5E). This may suggest that the sperm could be consuming more oxygen to maintain their velocity, as suggested by Rahi et al. [76]. This could compensate for the loss of sperm motility and thus help ensure fertilisation success. Similar to the observations at lower salinity, although no significant differences were observed (Figure 5F), the LIN appeared to decrease in sperm at higher salinity compared to that for non-contaminated sperm at control salinity (Figure 5F), potentially impairing fertilisation. Additionally, Eu 40 induced significantly more irregular sperm movement compared to that for the sperm at CTL 20 (Table 7), which can also impair fertilisation. Pagano et al. [77] found that in the sperm of *P. lividus*, 10^−5^ M of Eu did not induce any change in the success of fertilisation and the percentage of developmental defects in the offspring. On the other hand, 10^−4^ M of Eu caused a reduction in fertilisation success and an increase in the percentage of developmental defects. Cuccaro et al. [73] observed that the exposure of *F. enigmaticus* sperm to Cd and copper at a salinity of 40 did not induce any change in the production of ROS nor cause an increase in LPO levels in comparison to sperm kept at a salinity of 30.

### 3.3. Salinity of 20 vs. Salinity of 40

To compare the overall impacts of each treatment at salinities of 20 and 40 and identify the worst treatments for both adults and sperm, the Integrated Biological Index (IBR) was employed. This index is considered one of the most commonly used among similar indices developed in recent years, as noted by Catteau et al. [78]. Its wide applicability is demonstrated by its use by several authors, including Khan et al. [79] and Andrade et al. [41].

According to the IBR index, both elements combined with a salinity of 40 constituted the treatments that induced the most alterations in adult mussels (Figure 1B). Mussels exposed to these treatments exhibited inhibited SOD activity and increased CAT and GPx activities. Additionally, these mussels were under oxidative stress indicated by redox imbalance and higher LPO levels. For sperm, specific treatments also induced higher alterations. Pr 20 and Eu 40 induced the potential increase in the irregular movement of sperm. In addition, Eu 40 led to a reduction in motile sperm, while the sperm exposed to Pr 20 exhibited the lowest velocity and higher production of O_2_^−^. These alterations yielded the highest IBR scores for Pr 20 and Eu 40 in sperm, identifying them as the treatments most detrimental to sperm function in mussels (Figure 1B). Furthermore, comparing the IBR scores for adults and sperm provided insights into differential sensitivity. At lower salinity, the IBR scores for adults and sperm exposed to CTL and Eu were similar (Figure 1B), suggesting comparable sensitivity to these treatments. However, Pr at lower salinity seemed to induce higher alterations in sperm than in adults (Figure 1B), which could indicate a higher sensitivity of sperm to this treatment. Similar to the results for lower salinity, adults and sperm at higher salinity showed similar IBR scores when exposed to the CTL and Eu treatments (Figure 1B). On the other hand, contrary to what was observed at lower salinity, adult mussels exposed to Pr at higher salinity showed higher responsiveness than sperm, with a higher IBR score (Figure 1B). These findings highlight the complex interactions between environmental stressors like salinity and exposure to REEs, with salinity modulating the biological impacts of REEs on different life stages in mussels. The different sensitivities of adults and sperm to the tested stressors suggests that environments contaminated with REEs and subjected to fluctuating salinity could induce distinct ecological impacts on population dynamics and reproductive health.

These findings provide essential insights into how REEs and salinity shifts can impact aquatic ecosystems. These biochemical and histopathological alterations observed in mussels can impair cellular and physiological functions, energy allocation, and homeostasis, affecting their health, growth, and reproductive capacities. The observed effects on sperm further emphasise potential reductions in reproductive success, which could lead to a decline in mussel populations. A diminished mussel population could disrupt mussels’ ecological roles, such as maintaining trophic balance and supporting biodiversity. Additionally, it could reduce the critical ecosystem services provided by mussels, including water filtration and nutrient cycling, ultimately affecting the health and stability of the aquatic environment.

## 4. Conclusions

The present study demonstrated that shifts in salinity could modulate the effects of both Pr and Eu in different life stages of *Mytilus galloprovincialis*. High salinity combined with both elements induced the most alterations in adult mussels, including changes in defence mechanisms, causing redox imbalance and cellular damage. On the other hand, sperm showed particular sensitivity to specific REE–salinity combinations, with the most changes in motility and velocity observed when exposed to Pr at low salinity and Eu at higher salinity. Interestingly, while adults and sperm exhibited similar responses to Eu across salinities, Pr exposure revealed distinct vulnerabilities, with sperm being more affected at low salinity and adults being more affected at high salinity. These findings highlight the ecological risks posed by REE pollution, particularly under varying environmental salinities, which could influence mussel reproductive success and population dynamics. Such changes may impact ecosystems, affecting biodiversity and ecosystem services, including water filtration and nutrient cycling. This study emphasises the need for further research, incorporating additional REEs, species, and environmental stressors like temperature fluctuations or pH variations. Developing predictive models for REE toxicity under dynamic environmental scenarios is crucial for better risk assessment and mitigation strategies in coastal ecosystems prone to REE contamination. However, caution must be exercised when utilizing such models due to the complexity of REE-salinity interactions. This study highlights the need to validate models with observed data since half of the parameters were not additive for most treatments, and for one treatment in particular, neither half was additive. Additionally, this research provides some data that can help to predict potential ecological impacts induced by REEs–salinity interactions, helping to guide regulations aimed at protecting aquatic ecosystems and ensuring the sustainability of services provided by bivalves like mussels.

## Figures and Tables

**Table 1:** Comparison of Independent Action (IA) model predictions for the combination of praseodymium (Pr) and a salinity of 20. IA predictions not overlapping with the 95% confidence intervals of the observed results denote interactive effects. Interaction type is classified based on the magnitude of combined effects and direction of individual responses.

Endpoint	Predicted	Observed	95% CI Observed	Interaction Type (Pr 20)
ETS	0.071959	−0.09007	(−0.310–−0.130)	Additive
PROT	−0.50921	−0.38728	(−0.683–−0.092)	Additive
GLY	0.228956	−0.34281	(−0.650–−0.035)	One-directional synergism down
SOD	−0.74165	−0.6203	(−0.957–−0.283)	Additive
CAT	−0.10988	0.106398	(−0.351–0.564)	Additive
GPx	−1.24261	−0.86398	(−1.443–−0.285)	Additive
CbEs-*p*NPA	0.085179	−0.14267	(−0.276–−0.010)	Bidirectional synergism down
CbEs-*p*NPB	−0.37159	−0.09991	(−0.234–0.034)	One-directional antagonism down
GSTs	−0.62051	−0.37203	(−0.460–−0.284)	Bidirectional antagonism down
GSH/GSSG	0.436532	0.753512	(0.535–0.972)	Bidirectional synergism up
LPO adults	−1.42155	−0.51835	(−0.953–−0.083)	One-directional antagonism down
PC	−0.2615	−0.45842	(−0.622–−0.295)	One-directional synergism down
*I_h G_*	−1.02073	0.092166	(−0.267–0.451)	Bidirectional antagonism up
*I_h DT_*	0.630828	0.430727	(−0.137–0.998)	Additive
O_2_^−^	0.580274	0.58089	(0.557–0.605)	Additive
H_2_O_2_	−0.01442	−0.02047	(−0.124–0.083)	Additive
LPO sperm	−0.10185	−0.09611	(−0.115–−0.077)	Additive
% MOT	−0.24752	−0.28785	(−0.611–0.035)	Additive
VCL	−0.77113	−1.17094	(−1.518–−0.825)	One-directional synergism down
LIN	−2.03888	−0.94006	(−1.626–−0.254)	One-directional antagonism down
WOB	−0.06235	0.452257	(0.003–0.902)	Bidirectional synergism up

**Table 2:** Comparison of Independent Action (IA) model predictions for the combination of europium (Eu) and a salinity of 20. IA predictions not overlapping with the 95% confidence intervals of the observed results denote interactive effects. Interaction type is classified based on the magnitude of combined effects and direction of individual responses.

Endpoint	Predicted	Observed	95% CI Observed	Interaction Type (Eu 20)
ETS	0.109028	−0.23344	(−0.420–−0.047)	Bidirectional synergism down
PROT	−0.40301	−0.2228	(−0.367–−0.079)	One-directional antagonism down
GLY	0.32193	−0.45303	(−0.602–−0.304)	One-directional synergism down
SOD	−2.03106	−0.14184	(−0.556–0.272)	One-directional antagonism down
CAT	0.466841	−0.02106	(−0.227–0.185)	One-directional synergism down
GPx	−0.81864	−0.45428	(−0.876–−0.032)	Additive
CbEs-*p*NPA	−0.08488	−0.20759	(−0.446–0.031)	Additive
CbEs-*p*NPB	−0.52316	−0.24514	(−0.433–−0.058)	One-directional antagonism down
GSTs	−1.46789	−0.13932	(−0.323–0.45)	One-directional antagonism down
GSH/GSSG	0.605611	0.573314	(0.291–0.855)	Additive
LPO adults	−1.00502	−0.74099	(−1.092–−0.390)	Additive
PC	−0.83813	−0.30777	(−0.504–−0.112)	One-directional antagonism down
*I_h G_*	−0.77222	0.167188	(−0.253–0.587)	Bidirectional antagonism up
*I_h DT_*	0.550492	0.735764	(0.632–0.840)	One-directional synergism up
O_2_^−^	0.525968	0.521473	(0.445–0.598)	Additive
H_2_O_2_	0.112305	0.019767	(−0.193–0.233)	Additive
LPO sperm	−0.08337	−0.09313	(−0.242–0.055)	Additive
% MOT	−0.40977	−0.157	(−0.308–−0.006)	One-directional antagonism down
VCL	−0.49081	−0.46957	(−0.752–−0.187)	Additive
LIN	−1.51877	−1.06139	(−1.925–−0.198)	Additive
WOB	−0.03455	0.078448	(−0.115–0.272)	Additive

**Table 3:** Comparison of Independent Action (IA) model predictions for the combination of praseodymium (Pr) and a salinity of 40. IA predictions not overlapping with the 95% confidence intervals of the observed results denote interactive effects. Interaction type is classified based on the magnitude of combined effects and direction of individual responses.

Endpoint	Predicted	Observed	95% CI Observed	Interaction Type (Pr 40)
ETS	−0.33829	−0.19463	(−0.438–0.049)	Additive
PROT	−0.15784	−0.01133	(−0.086–0.064)	Bidirectional antagonism down
GLY	0.509975	0.253243	(0.054–0.453)	One-directional antagonism up
SOD	−1.96644	−2.04563	(−2.535–−1.556)	Additive
CAT	0.209357	0.548691	(0.483–0.614)	Bidirectional synergism up
GPx	−0.27923	0.114762	(−0.054–0.284)	Bidirectional synergism up
CbEs-*p*NPA	−0.19717	−0.43836	(−0.511–−0.366)	One-directional synergism down
CbEs-*p*NPB	−0.9087	−0.70487	(−0.817–−0.592)	One-directional antagonism down
GSTs	−0.802	−0.58439	(−0.767–−0.402)	Bidirectional antagonism down
GSH/GSSG	−0.86958	−0.19044	(−0.656–0.275)	One-directional antagonism down
LPO adults	−0.29403	1.031288	(0.861–1.201)	Bidirectional synergism up
PC	−0.0718	−0.20163	(−0.424–0.021)	Additive
*I_h G_*	−0.18414	−0.06964	(−0.184–0.044)	Bidirectional antagonism up
*I_h DT_*	0.718506	1.022093	(0.702–1.343)	Additive
O_2_^−^	0.18894	−0.0328	(−0.116–0.051)	One-directional synergism up
H_2_O_2_	−0.01296	−0.08133	(−0.386–0.224)	Additive
LPO sperm	−0.13838	−0.13397	(−0.229–−0.039)	Additive
% MOT	−0.07212	−0.14695	(−0.439–0.145)	Additive
VCL	0.101054	0.137288	(−0.060–0.334)	Additive
LIN	−2.41146	−1.11216	(−2.273–−0.049)	One-directional antagonism down
WOB	−0.33343	−0.04048	(−0.301–0.220)	One-directional antagonism down

**Table 4:** Comparison of Independent Action (IA) model predictions for the combination of europium (Eu) and a salinity of 40. IA predictions not overlapping with the 95% confidence intervals of the observed results denote interactive effects. Interaction type is classified based on the magnitude of combined effects and direction of individual responses.

Endpoint	Predicted	Observed	95% CI Observed	Interaction Type (Eu 40)
ETS	−0.30122	−0.18343	(−0.390–0.023)	Additive
PROT	−0.05164	0.186069	(0.090–0.282)	Bidirectional synergism up
GLY	0.602949	0.459562	(0.364–0.555)	One-directional antagonism up
SOD	−3.25585	−1.02806	(−1.498–−0.558)	One-directional antagonism down
CAT	0.786073	0.701732	(0.523–0.881)	Additive
GPx	0.144731	0.279154	(0.073–0.485)	Additive
CbEs-*p*NPA	−0.36723	−0.07788	(−0.193–0.037)	One-directional antagonism down
CbEs-*p*NPB	−1.06027	−0.47392	(−0.673–0.275)	One-directional antagonism down
GSTs	−1.64939	−0.78145	(−1.373–−0.190)	One-directional antagonism down
GSH/GSSG	−0.7005	−0.98813	(−1.298–−0.678)	Additive
LPO adults	0.122501	0.890802	(0.586–−1.195)	Bidirectional synergism up
PC	−0.64843	−0.07429	(−0.304–0.156)	Bidirectional antagonism down
*I_h G_*	0.064371	0.347344	(−0.104–0.799)	Additive
*I_h DT_*	0.63817	0.774256	(0.601–0.947)	Additive
O_2_^−^	0.134634	0.117904	(−0.021–0.257)	Additive
H_2_O_2_	0.11376	−0.214	(−0.604–0.176)	Additive
LPO sperm	−0.1199	−0.12805	(−0.192–−0.064)	Additive
% MOT	−0.23437	−0.8684	(−1.169–−0.568)	One-directional synergism down
VCL	0.381371	0.259969	(−0.471–0.991)	Additive
LIN	−1.89135	−0.82537	(−1.732–0.081)	One-directional antagonism down
WOB	−0.30564	0.308756	(−0.035–0.653)	One-directional synergism up

**Table 5:** Praseodymium (Pr) and europium (Eu) concentrations in water samples (μg/L) collected after spiking from exposure aquaria as well as in mussel whole soft tissue (μg/g dry weight (DW)) and BCF (L/Kg) after 28 days of exposure to CTL, Pr, and Eu at salinities of 20, 30, and 40. Results are presented as the mean ± standard deviation. Significant differences (*p* < 0.05) among salinities are denoted by different letters (bold, italic lowercase letters indicate CTL treatments; uppercase letters indicate Pr treatments; and lowercase letters indicate Eu treatments).

		Water Samples (µg/L)		Tissue Samples (µg/g DW)		BCF (L/Kg)	
		Pr	Eu	Pr	Eu	Pr	Eu
Sal 20	CTL	<0.01	<0.01	0.003 ± 0.001 ***^a^***	<0.03 ***^a^***	-	-
	Pr	12 ± 0.5	-	0.6 ± 0.1 ^A^	-	49	-
	Eu	-	13 ± 0.4	-	0.2 ± 0.06 ^a^	-	18
Sal 30	CTL	<0.01	<0.01	0.008 ± 0.004 ***^ab^***	<0.03 ***^a^***	-	-
	Pr	13 ± 0.8	-	0.3 ± 0.04 ^B^	-	25	-
	Eu	-	14 ± 0.5	-	0.2 ± 0.02 ^a^	-	15
Sal 40	CTL	<0.01	<0.01	0.007 ± 0.001 ***^b^***	<0.03 ***^a^***	-	-
	Pr	13 ± 0.5	-	0.2 ± 0.09 ^B^	-	19	-
	Eu	-	14 ± 0.7	-	0.2 ± 0.07 ^a^	-	14

**Table 6:** Praseodymium (Pr) and europium (Eu) concentrations (μg/L) in seawater samples collected from blanks in the first week (0, 72, 120, and 168 h after spiking) at three different salinities. Results are the mean ± standard deviation.

		Water Sample Concentrations (µg/L)			
		0 h	72 h	120 h	168 h
Sal 20	Pr	13 ± 0.09	11 ± 0.2	11 ± 0.04	9.4 ± 0.07
	Eu	13 ± 0.4	13 ± 0.6	12 ± 0.4	12 ± 0.9
Sal 30	Pr	13 ± 0.03	11 ± 0.3	11 ± 0.4	9.3 ± 0.3
	Eu	14 ± 0.6	12 ± 0.4	11 ± 0.07	13 ± 1.6
Sal 40	Pr	13 ± 0.3	11 ± 0.4	9.1 ± 0.3	9.9 ± 0.7
	Eu	14 ± 0.3	12 ± 0.1	11 ± 0.2	10 ± 0.3

**Table 7:** Comparison among CTL, Pr, and Eu at each salinity along with the *p*-values. For each parameter/histopathological index (*I_h_*), significant differences (*p* < 0.05) between treatments are denoted by the *p*-values in bold.

Parameter/*I_h_*	Salinity 20			Salinity 30			Salinity 40		
	CTL vs. Pr	CTL vs. Eu	Pr vs. Eu	CTL vs. Pr	CTL vs. Eu	Pr vs. Eu	CTL vs. Pr	CTL vs. Eu	Pr vs. Eu
[Pr] tissue	**0.0004**	-	-	**0.0002**	-	-	**0.0079**	-	-
[Eu] tissue	-	**0.0025**	-	-	**0.0001**	-	-	**0.0114**	-
ETS	**0.040**	**0.013**	0.29	**0.045**	0.18	0.69	0.66	0.68	0.95
GLY	**0.025**	**0.0068**	0.43	0.31	**0.033**	0.21	0.14	0.55	0.074
PROT	0.40	0.72	0.30	**0.0057**	0.17	0.32	0.28	0.37	**0.017**
SOD	0.68	0.14	0.11	0.55	**0.030**	**0.0080**	0.32	0.46	**0.023**
CAT	0.99	0.14	0.50	0.20	**0.037**	**0.015**	0.33	0.088	0.13
GPx	0.71	0.11	0.21	0.10	0.53	0.14	0.25	0.053	0.20
CbEs-*p*NPA	**0.014**	**0.026**	0.62	0.20	0.051	0.11	0.053	0.85	**0.0047**
CbEs-*p*NPB	0.42	0.095	0.20	**0.0047**	**0.023**	0.39	0.47	0.80	0.068
GSTs	0.25	0.077	0.56	0.51	**0.011**	**0.010**	0.20	0.73	0.50
GSH/GGSG	**0.049**	0.38	0.26	0.90	0.50	0.50	0.067	0.52	**0.034**
LPO adults	0.38	0.87	0.38	0.10	0.50	0.15	**0.015**	0.053	0.38
PC	0.055	0.32	0.22	0.29	0.062	0.11	0.083	0.49	0.37
*I_h_* Gills	**0.024**	**0.022**	0.74	0.75	0.25	0.28	**0.016**	**0.047**	0.11
*I_h_* Digestive tubules	0.30	0.059	0.25	**0.024**	**0.025**	0.56	**0.0079**	**0.0045**	0.19
O_2_^−^	**0.046**	0.36	0.14	0.42	0.45	**0.043**	0.056	0.62	0.091
H_2_O_2_	*	*	*	*	*	*	*	*	*
LPO sperm	0.58	0.87	0.93	0.23	0.86	0.43	0.69	0.70	0.92
% MOT	0.85	0.16	0.44	0.44	0.36	0.16	0.96	**0.0057**	**0.014**
VCL	**0.016**	0.55	**0.018**	0.68	0.70	0.51	0.29	0.97	0.59
LIN	*	*	*	*	*	*	*	*	*
WOB	0.16	0.97	0.16	0.24	0.61	0.80	0.26	**0.036**	0.11

* Main test > 0.05.

## Data Availability

The data presented in this study are available on request from the corresponding author. (as authors wish to be informed of their use).
